# An Analysis of Rheumatoid Arthritis Hospitalizations

**DOI:** 10.7759/cureus.12344

**Published:** 2020-12-28

**Authors:** Sandhya Shri Kannayiram, Armaan Guraya, Chukwudi C Muojieje, Karun M Nair, Osahon N Idolor, Jesse Odion, Osaigbokan P Aihie, Eseosa Sanwo

**Affiliations:** 1 Internal Medicine, John H. Stroger, Jr. Hospital of Cook County, Chicago, USA; 2 Medicine, Midwestern University Chicago College of Osteopathic Medicine, Downers Grove, USA; 3 Internal Medicine, Mountain View Regional Medical Center, Las Cruces, USA; 4 Medicine, University of Benin College of Medicine, Benin City, NGA; 5 Internal Medicine, University of Benin Teaching Hospital, Benin City, NGA; 6 Medicine, School of Medicine, University of Missouri, Columbia, USA

**Keywords:** : rheumatoid arthritis, hospitalization, sepsis, infection, cardiovascular, mortality, national inpatient sample

## Abstract

Background

We used a large United States (US) population-based database to analyze the reasons for hospitalization of rheumatoid arthritis (RA) patients.

Methods

The International Classification of Diseases, Tenth Revision (ICD-10) code was used to search for hospitalizations in 2017 in the National Inpatient Sample (NIS) database with RA as the principal or secondary diagnosis. The reasons for hospitalization were divided into 19 categories based on their principal discharge ICD-10 diagnosis code. We also ranked the five most common specific reasons for hospitalization.

Results

There were over 35 million discharges included in the 2017 NIS database; 565,440 hospitalizations had either a principal or secondary ICD-10 code for RA. The top five reasons for RA hospitalization by ICD-10 code categories were as follows: cardiovascular (CV): 93,825 (16.59%), rheumatologic: 82,785 (14.64%), respiratory: 66,895 (11.83%), infection: 62,660 (11.09%), and injury/poisoning: 56,460 (9.96%). Sepsis was the most common principal diagnosis for RA hospitalizations.

Conclusion

CV diseases were the most common ICD category, and sepsis was the most common principal diagnosis for RA hospitalizations. Management of medical comorbidities (such as CV) and prevention of infection is essential for reducing the rates of RA hospitalizations.

## Introduction

Rheumatoid arthritis (RA) is a chronic autoimmune disorder that mainly affects the synovial joints, affecting up to 0.24-1% of the global population. It is one of the most common inflammatory disorders, with a clear predilection towards the female gender, occurring at a ratio of 2.5:1 compared with men [1]. It manifests in varying frequencies across different countries and different ethnic groups, being more common in the United States (US) and northern Europe than Southern Europe and countries in Asia and Africa [2]. This reported difference in prevalence among different countries and ethnic groups has been attributed to genetic, environmental, and lifestyle factors as well as a lack of enough studies in developing countries. Although studies have shown that the incidence of RA is decreasing in countries with high prevalence, RA continues to be one of the major contributors to the global disease and disability burden [3,4].

RA has a significant impact on health-related quality of life, causing loss of functional capacity and disability in the long term [5]. It affects physical and mental well-being and is associated with an increased risk of poor sleep quality, anxiety, depression, and excessive fatigue [6]. Apart from affecting mental and physical health, RA also causes a substantial economic burden to the patients, their caregivers, hospitals, and society due to the loss of work capacity, poor quality of life, multiple comorbidities, and premature mortality associated with the disease [7].

RA, being a multisystemic inflammatory disease, is associated with extra-articular manifestations such as coronary artery disease, interstitial lung disease, anemia, stroke, peripheral vascular disease, and increased risk of thromboembolism, particularly with severity and longer duration of disease [8]. The disease by itself and the use of immunosuppressive agents for its treatment have caused an increased prevalence of serious comorbidities like osteoporosis, infections, renal disease, and lymphoproliferative disorders in patients with RA [9]. Severe RA is associated with decreased life expectancy, increased risk of hospitalizations, and a two-fold increase in mortality [10]. Early diagnosis and recognition of RA and its comorbidities and a multi-modality approach to treatment are essential to decrease the mortality rates associated with the disease.

Over the last decade, a few population-based studies have demonstrated decreasing mortality and hospitalizations among RA patients [11]. Furthermore, only limited national-level data is available in the US on the reasons for hospitalizations of RA patients. Hence, in this report, we used the National Inpatient Sample (NIS), a large US population-based database, to address this clinically relevant issue.

## Materials and methods

Data source

The NIS was used to search for hospitalizations in 2017 with the International Classification of Diseases, Tenth Revision (ICD-10) RA codes "M05" and “M06” as the principal or secondary diagnosis. NIS is the largest hospitalization-related database in the US [12-15]. It entails a 20% probability sampling of different strata, which is structured to be representative of hospitalizations at the national level [16-19]. Each hospitalization in the 2017 NIS has a principal diagnosis and up to 40 secondary diagnoses. The principal diagnosis is the main reason for hospitalization, while the remaining diagnoses are secondary diagnoses [20,21]. Since patient data in NIS is de-identified, and the NIS database is available to the public, this study was exempted from the institutional review board approval.

Inclusion and exclusion criteria

The study population consisted of all hospitalizations in 2017 with a principal or secondary RA ICD-10 diagnosis codes. We excluded hospitalizations for patients who were younger than 18 years. We used ICD-10 codes “M05” and “M06” to identify hospitalizations with a principal or secondary diagnosis of RA.

Statistical analysis

All statistical analyses were performed using the Stata software, version 16.

Outcomes

The total number of RA discharges, age, race, length of stay (LOS), and total hospital charges incurred were recorded. The reasons for hospitalization were divided into 19 categories based on their principal discharge ICD-10 diagnosis codes. We also ranked the five most common specific reasons for hospitalization. The principal diagnosis for hospitalization was considered to be the reason for hospitalization.

## Results

There were over 35 million discharges included in the 2017 NIS database; 565,440 hospitalizations were for patients aged 18 years or above, who had either a principal or secondary ICD 10 code for RA. Patients were predominantly female (74%), White (65%), with an average age of 67 years. The average LOS was five days and the average total hospital charge was $57,418. The top five reasons for hospitalization for adult RA patients by ICD 10 code categories in descending order of frequency were as follows (Table 1): cardiovascular (CV): 93,825 (16.59%), rheumatologic: 82,785 (14.64%), respiratory: 66,895 (11.83%), infection: 62,660 (11.09%), and injury/poisoning: 56,460 (9.96%). Sepsis was the most common principal diagnosis followed by acute chronic obstructive pulmonary disease (COPD) exacerbation, pneumonia, osteoarthritis of the right knee, acute kidney injury (AKI) in descending order of frequency. RA was the 25th most common principal discharge diagnosis.

**Table 1:** ICD-10 code categories for hospitalizations of rheumatoid arthritis patients ICD-10: the International Classification of Diseases, Tenth Revision

ICD-10 Code Admission Category	Number of Admissions
Certain infections and parasitic diseases	62,660
Neoplasms and diseases of the blood and blood-forming organs and certain disorders involving the immune mechanism	24,530
Endocrine, nutritional, and metabolic diseases	20,765
Mental, behavioral, and neurodevelopmental disorders	12,510
Diseases of the nervous system	13,820
Diseases of the eye and adnexa and ear	1,090
Diseases of the circulatory system	93,825
Diseases of the respiratory system	66,895
Diseases of the digestive system	56,270
Diseases of the skin and subcutaneous tissue	15,175
Diseases of the musculoskeletal system and connective tissue	82,785
Diseases of the genitourinary system	28,930
Pregnancy, childbirth, and puerperium	4,875
Certain conditions originating in the perinatal period	0
Congenital malformations, deformations, and chromosomal abnormalities	420
Symptoms, signs, and abnormal clinical laboratory findings, not classified elsewhere	18,820
Injury, poisoning, and certain other consequences of external causes	56,460
External causes of morbidity (accidents and violence)	10
Factors influencing health status and contact with health services	5,595

## Discussion

In order to shed light on the morbidity rate of patients with RA on a national level, we conducted a comprehensive review of the NIS database. The major findings of our study are as follows: i) the most common reason for hospitalization for adult RA patients as per ICD-10 code categories was CV, with sepsis as the most common principal diagnosis, and ii) rheumatologic disorders were the second most common ICD category for hospitalization; however, RA was the 25th most common principal diagnosis for hospitalization.

We found CV diseases to be the most common cause of hospitalization in RA patients, which is in line with other studies [22,23]. This finding is consistent with what is known about the increased CV burden associated with RA in the absence of other risk factors. Nonetheless, this further emphasizes the need to optimize preventative CV practices in a patient population that is commonly overlooked as being high risk for CV diseases. Proper management in the primary care setting may prevent a sizeable number of hospitalizations among RA patients. Further studies are needed to clarify how aggressive an approach is required and whether prophylactic pharmaceutical intervention is warranted. There is prudence in the early collaboration between the rheumatologist and cardiologist.

Infection was the fourth most common ICD-10 code category; however, sepsis was the most common specific principal diagnosis for RA hospitalizations. The Institute of Rheumatology, Rheumatoid Arthritis (IORRA) cohort was found to have infections as the most common cause of hospitalized comorbidity, while another single-center study found infections to be the leading cause of death in RA patients admitted to their ICU [24,25]. Similarly, Other studies have found RA to be an independent risk factor for long-term mortality due to ICU admission with sepsis [26]. These findings speak to the importance of early detection of infections that can lead to sepsis because its existence leads to dire consequences in all patients. While complications of the aforementioned CV disease are of a more chronic nature, infectious sequelae require acute detection and remediation. RA patients may need a lower threshold of suspicion given that they may be on immunosuppressive therapies and should be identified and treated early on.

Rheumatologic disorders were the second most common ICD category for hospitalization; however, RA was the 25th most common principal diagnosis for hospitalization. This discrepancy underlines the utility in addressing the many comorbidities of RA: the disease may not drive hospitalizations, but patients’ susceptibility to other diseases, both rheumatological and not, do. A multidisciplinary approach is necessary to reduce rates of hospitalization, and this may include patient education, as a study at a tertiary care hospital found only 1.5% of RA patients are “aware” of their disease [27]. The relationship between patient and physician must be optimized by providing patients with the tools of pharmacotherapy, lifestyle changes, and education, and by empowering the physician by raising their clinical suspicion for the detection and prevention of RA comorbidities.

The large sample size and nature of the NIS, which enabled us to categorize reasons for hospitalizations into different ICD-10 organ-system-based categories and specific reasons are some of the strengths of this study. However, there are some limitations to our study: i) the NIS is an administrative database that uses claims data via ICD-10 codes to characterize diagnoses, and hence there is a possibility of errors being associated with coding [28]; ii) this report reflects data on RA hospitalizations rather than on individual patients [29]; iii) most ICD-10 codes do not grade disease severity, and NIS does not contain information on medication compliance [30]. Hence, we could not determine if underlying RA severity, and immunosuppressant compliance may have affected the number of RA hospitalizations.

## Conclusions

For adult patients with a history of RA, CV diseases were the most common ICD category for hospitalization, while sepsis was the most common principal diagnosis for hospitalization. Rheumatologic disorders were the second most common ICD category for hospitalization; however, RA was the 25th most common principal diagnosis for hospitalization. Though RA patients are likely to be admitted because of other rheumatologic disorders, they are far less likely to be admitted principally because of RA. Hence adequate management of medical comorbidities (e.g., CV and respiratory disorders) is needed to reduce the rate of hospitalization.
